# Community Pharmacists’ Role in Reducing the Incidence of Cardiometabolic Adverse Drug Events in Schizophrenia: Insights from Mental Health Professionals

**DOI:** 10.3390/medicina59122052

**Published:** 2023-11-21

**Authors:** Rahnee A. Karge, Colin M. Curtain, Mohammed S. Salahudeen

**Affiliations:** School of Pharmacy and Pharmacology, College of Health and Medicine, University of Tasmania, Hobart, TAS 7005, Australia; rakarge@utas.edu.au (R.A.K.); colin.curtain@utas.edu.au (C.M.C.)

**Keywords:** schizophrenia, community pharmacists, cardiometabolic, mental health, qualitative study

## Abstract

*Background and Objectives:* Schizophrenia, a debilitating mental illness, is often associated with significant physical health risks. Many second-generation antipsychotics increase the risk of metabolic syndrome and cardiovascular disease. Community pharmacists are highly accessible and could play a role in monitoring cardiometabolic adverse drug events in people with schizophrenia. However, it remains uncertain whether mental health professionals perceive this as valuable. This study aimed to explore the opinions of mental healthcare professionals regarding the role of community pharmacists in reducing the incidence of cardiometabolic adverse events in people with schizophrenia and their integration into a multidisciplinary mental health team. *Materials and Methods:* Qualitative semi-structured interviews were conducted with Australian psychiatrists, mental health nurses and mental health pharmacists. Transcription of the interviews underwent thematic analysis using an inductive approach. *Results:* Eleven mental healthcare professionals from metropolitan and regional areas across Australia were interviewed, leading to the identification of five overarching themes. These themes encompassed the following aspects: the benefits of community pharmacists’ involvement in managing cardiometabolic adverse drug events in people with schizophrenia, improving communication pathways with community pharmacists, defining roles and responsibilities for monitoring cardiometabolic parameters and managing adverse cardiometabolic drug events, fostering collaboration between community pharmacists and mental health care professionals, and recognising the acceptance of community pharmacists’ integration within a multidisciplinary team. Mental health professionals believed that community pharmacists could play a role in reducing the incidence of cardiometabolic adverse events in schizophrenia. However, they underscored the need for enhanced communication and collaboration pathways with other healthcare professionals, emphasised the importance of more comprehensive mental health first aid training, and identified potential barriers for community pharmacists such as remuneration, workload, and staff resources. *Conclusions:* Mental health professionals acknowledged the benefits of incorporating community pharmacists into multidisciplinary teams as a strategy to reduce the incidence of adverse events among individuals with schizophrenia. They recognise the competence of community pharmacists in monitoring cardiometabolic adverse events. However, these professionals have also highlighted specific perceived barriers to the complete integration of community pharmacists within these teams. Notably, there are concerns related to remuneration, staff resources, time constraints, acceptance by other healthcare professionals and patients, and the need for improved communication pathways. Addressing these barriers and providing targeted training could facilitate the valuable inclusion of community pharmacists in the comprehensive care of people with schizophrenia.

## 1. Introduction

Schizophrenia is a severe mental illness that affects approximately 1% of the world population, with symptoms including delusions, hallucinations, cognitive impairment, and difficulties with social and emotional functioning [1]. People with schizophrenia have a life expectancy of 10 to 15 years less than the general population, and an all-cause mortality rate 2 to 3 times higher [2,3,4]. Although suicide is a prominent cause of death, individuals with schizophrenia are twice as likely to develop type II diabetes (T2D) and cardiovascular disease (CVD) compared to the general population, with approximately one-quarter of mortality in individuals with schizophrenia attributed to CVD [5].

Though lifestyle factors and genetic predisposition may contribute to this risk, many second-generation antipsychotics cause weight gain and contribute to dyslipidaemia and impaired glucose tolerance, with metabolic changes occurring as early as three months after initiation [6,7,8,9]. The International Diabetes Federation defines metabolic syndrome as central obesity accompanied by at least 2 of the following: elevated triglycerides, reduced high-density lipoprotein, hypertension, or raised fasting blood glucose [10]. People with schizophrenia have a 2–3 times higher risk of developing metabolic syndrome, a strong predictor of CVD and T2D [11,12].

Mental healthcare professionals are aware of the importance of routine metabolic syndrome risk factor monitoring for people with schizophrenia, including weight and body mass index (BMI) calculation, blood pressure, waist circumference, and lipid and glycaemic profile monitoring. Despite this, comprehensive cardiometabolic parameter monitoring is estimated to occur in less than 30% of patients [13]. The inclusion of specialist mental health pharmacists in a general practice setting has been shown to increase the screening of metabolic parameters in people with schizophrenia, improve quality of life, enhance continuity between primary and secondary care, and ultimately improve patient outcomes [14,15].

The necessity for enhanced adverse drug event (ADE) monitoring among mental health patients in the community has been highlighted, however, there remains a lack of clarity regarding the entity responsible [16]. In addition, the frequency and extent of cardiometabolic parameter monitoring continue to be inadequate when compared to existing guidelines, with studies reporting barriers including interference with patient rapport, excessive time and effort involved, the belief that routine monitoring is unnecessary for all patients, and lack of access to resources such as scales, height charts, and blood pressure cuffs [17].

Community pharmacists are highly accessible, with 5822 community pharmacies in Australia in 2020 [18]. At present, there is insufficient evidence to evaluate the clinical outcomes of community pharmacists’ intervention in cardiometabolic ADEs in people with schizophrenia [19]. Community pharmacists could play a beneficial role in preventing and monitoring cardiometabolic adverse drug events in people with schizophrenia. Whether this role would be considered valuable by mental healthcare professionals such as psychiatrists, mental health nurses, and mental health pharmacists is unclear. Hence, the aim of this study was to explore mental healthcare professionals’ opinions regarding the integration of community pharmacists within a multidisciplinary mental health team, with the purpose of reducing the incidence of cardiometabolic adverse drug events, such as the development of metabolic syndrome, in people with schizophrenia.

## 2. Materials and Methods

A qualitative thematic analysis following an inductive approach was used to uncover mental health professionals’ perspectives, attitudes, and beliefs on the role of community pharmacists in monitoring and managing cardiometabolic adverse events in people with schizophrenia. Thematic analysis was based on the 6 phases of thematic analysis by Braun and Clarke [20]. Interview questions identified previous interactions with community pharmacists regarding people with schizophrenia, the perceived capabilities of community pharmacists in monitoring cardiometabolic parameters, participants’ opinions on the benefits, and suggested barriers to community pharmacists’ integration within a multidisciplinary mental health team. Ethics approval was obtained from the University of Tasmania Human Research Ethics Committee (HREC27994).

Psychiatrists, mental health nurses and mental health pharmacists, currently registered in Australia with at least one year of experience working with people with schizophrenia, were recruited via advertisements on social media platforms (LinkedIn, Facebook) and electronic newsletters from “The Australian College of Mental Health Nurses”, and “SHPA—The Society of Hospital Pharmacists of Australia—Mental Health Interest Group”. Pharmacists who worked as community pharmacists, as well as in a mental health setting, were excluded. Interested participants were emailed a consent form and information sheet, and asked to provide demographic data, including gender, age distribution, profession, years of experience working in mental health and location of practice. Written informed consent was sought prior to participation and verbally confirmed at the commencement of the interview.

The interviews were conducted via the Zoom videoconferencing software between November 2022 and May 2023. A semi-structured approach with open and expansive questions was used to identify participant’s past experiences with community pharmacists’ involvement in the management of people with schizophrenia, their thoughts on community pharmacists’ capabilities, whether they believed there was a benefit in including community pharmacists in a multidisciplinary team, and any perceived barriers to this (Supplementary File S1). The interviews were concluded with the opportunity for the participant to discuss any of the covered themes further. The audio of the interviews was recorded and then transcribed using Microsoft Word. The transcripts were reviewed manually by the researchers (RK, MS, CC). Participants were given a chance to review the transcript within the week before de-identification, to ensure their views were accurately represented. Participants were informed that they could withdraw consent at any time during the interview and prior to data de-identification.

Thematic analysis employed the qualitative data analysis software QualCoder version 3.2 (https://github.com/ccbogel/QualCoder/releases/tag/3.2) to develop codes and categories [21]. These were discussed and revised by researchers (RK, CC) for validation, with a cyclic process to create the final themes [20]. It was agreed that saturation of themes was achieved after 11 interviews.

## 3. Results

Thirteen participants were recruited, of which 11 were interviewed, comprising 5 mental health pharmacists, 3 mental health nurses, and 3 psychiatrists. Most participants (82%) were female, with 7 working in metropolitan and 4 in regional areas. The majority (73%) had at least 16 or more years of experience, and the mean interview duration was 29 min. Participant demographics are shown in Table 1.

Five themes were developed to elucidate mental healthcare professionals’ views on the integration of community pharmacists within a multidisciplinary mental health team to monitor and reduce the incidence of cardiometabolic adverse drug events in people with schizophrenia. Deductive thematic saturation was achieved with the consistency of data generated, and no new insights were identified beyond interview 9. These themes are described below, with illustrative quotations and the participant’s unique identifier. Themes and sub-themes are summarised in Table 2.

### 3.1. Theme 1: The Benefits of Community Pharmacists’ Involvement in the Management of Cardiometabolic Adverse Drug Events in People with Schizophrenia

Participants stated that community pharmacists currently have a beneficial role in the management of people with schizophrenia via professional services such as dose administration aids, clozapine dispensing, medication compliance, medication information, and providing dispensing histories when requested by mental health professionals.

*“…patients with schizophrenia often can have subtle cognitive issues over time that can sort of mean that a single long, detailed conversation with the doctor might not be absorbed… questions come up that don’t get addressed in the session, so I think there’s a big role for them in terms of maintaining compliance.”*

(Psychiatrist 10)

Participants expressed the beneficial positive rapport that community pharmacists have with their patients.

*“I get very helpful feedback about how the patient’s going from the team in the pharmacy, because they know the patient and they’re like, yeah, we think that they’re pretty much struggling right now.”*

(Pharmacist 11)

Community pharmacy accessibility was beneficial when considering the monitoring of cardiometabolic parameters.

*“There are so many occasions when our clientele cannot get to a GP [General Pracitioner]. GPs are harder and harder to get to, they’re not available… they might be full up and you can’t get an appointment for three weeks, or they only work part-time.”*

(Nurse 7)

*“When I think about it, you know it’s very acceptable to patients, which is probably a key thing with people with schizophrenia, maybe that’s the biggest thing, how acceptable it is and how accessible, it’s near their home.”*

(Pharmacist 2)

Participants suggested the services community pharmacists provide for people with schizophrenia could be extended.

*“…not that the patients can go there to get their depots and things, it’s a bit outside of the scope of practice, though I think, that would be a really good service too if they could administer depots.”*

(Psychiatrist 4)

*“Well, they do vaccinations and all that sort of stuff, so it’s definitely a field that they can get into, to actually perform the pathology themselves”.*

(Pharmacist 1)

### 3.2. Theme 2: Improving Communication Pathways with Community Pharmacists

Although participants suggested that community pharmacists have the competency and many resources for monitoring cardiometabolic parameters in people with schizophrenia, communication pathways with mental health professionals were suggested to be currently insufficient.

*“Just better [communication] systems that everyone can access, like community and hospitals and community mental health clinics. Every place that the patient is involved within their care, the pharmacist and all of those places being able to have the same level of access.”*

(Pharmacist 1)

Under-utilization of My Health Record (MHR), an Australia-wide electronic health record system, as a communication platform was described.

*“I was hoping my health record would be better utilized by community pharmacies than it is.”*

(Pharmacist 1)

However, it was suggested that My Health Record may not contain all the relevant information for community pharmacists, and access to legal records may be required when monitoring a patient with schizophrenia.

*“We also manage patients with forensic backgrounds or physical or violent backgrounds, and you know, our psychiatrists and our primary clinicians have really got understanding of their background and what their safety profile is… We have access to those notes [legal]. We review those notes before we interact with these people, so we understand those risks and a community pharmacist, without access to the sort of case history, might not be aware of the risks.”*

(Pharmacist 2)

### 3.3. Theme 3: Defining the Role and Responsibility for Monitoring Cardiometabolic Parameters and Managing Adverse Cardiometabolic Drug Events

Participants highlighted that there is currently no consensus over which healthcare professionals are responsible for monitoring cardiometabolic parameters, with the onus often falling on allied health staff.

*“There’s always so many patients and, everyone’s got an idea that metabolic monitoring needs to occur, but there’s kind of a bit of debate about whose responsibility it is. Like the public health care providers think that the GPs can do it, and the GPs don’t always have time, so pharmacies would be well placed to support that whole system.”*

(Pharmacist 1)

*“…honestly, I think that that would be a huge relief to the social workers, nurses and other case managers that are there… they have to chase up all of these things, and if there was somebody that was trained in that area, and able to take on that, then I think that that would be very popular.”*

(Psychiatrist 4)

When it came to the responsibility for the management of cardiometabolic adverse events, mental healthcare professionals suggested that this, too, was contentious.

*“We’re only meant to prescribe the depot and clozapine, the GP is meant to manage all the other orals, but often we might do the orals of mental health drugs, but certainly, with the other conditions… there is a misconception amongst a lot of our clients that, oh they see us, they don’t need to see other doctors. As you know, that’s not the case, it’s very important that they see their GP so that they can have their other comorbidities managed.”*

(Pharmacist 2)

Participants suggested that community pharmacists’ monitoring of cardiometabolic parameters may help with early intervention of cardiometabolic adverse events.

*“I forgot to talk about the incredible role pharmacists could have with getting metformin commenced in a timely fashion, as well as statins and blood pressure management.”*

(Pharmacist 11)

It was recommended that further training in mental health would be important for community pharmacists to be included in a multidisciplinary mental health team.

*“… some formal mental health information and training would be ideal, even Mental Health First Aid.”*

(Nurse 7)

*“You might even need to run a course for six months or something like that.”*

(Pharmacist 1)

### 3.4. Theme 4: Fostering Collaboration between Community Pharmacists and Mental Health Care Professionals

Participants suggested that there were advantages to collaboration with community pharmacists for cardiometabolic parameter monitoring.

*“I think actually a lot of community mental health teams would love it if the pharmacist would just do that, and talk to the GP, because we have a lot of community mental health teams that don’t have many nurses… they’re often social workers or psychologists who don’t have this background, and it’s sort of is outside their scope of practice.”*

(Pharmacist 8)

Regarding cardiometabolic parameter monitoring, participants suggested that a collaborative approach with general practitioners was important.

*“…they need to do it in collaboration with the GPs and if it doesn’t work, happen collaboratively, it would never be successful…. so yeah, I think they’re well placed, but it does require a collaborative relationship all around.”*

(Pharmacist 1)

The need for a well-defined pathway to feedback metabolic results was suggested so that the responsibility for follow-up did not sit solely with patients.

*“A really good algorithm too, for what to do and who to refer to, and who to let know to make sure that if there are alarm bells, … that that’s appropriately referred”.*

(Psychiatrist 10)

*“Where are you going to send your results, or who are you going to notify, and then it can be a bit frustrating because despite telling someone that you know that they’ve high blood pressure, their lipids are raised… depending on the patient, you can’t necessarily actually get them to do something about it. And it’s typically a kind of population that don’t often go to a GP.”*

(Pharmacist 3)

### 3.5. Theme 5: Recognizing the Acceptance of Community Pharmacists’ Involvement in a Multidisciplinary Team

A role for community pharmacists’ involvement as part of a multidisciplinary team, needs to be acceptable to the community pharmacist. Remuneration, lack of time and space constraints, were seen as barriers to community pharmacists’ involvement.

*“One of the barriers is that they have a busy schedule, there are a lot of people coming in for various different conditions, and schizophrenia is just one of the many conditions…so I think time constraints is the big limitation.”*

(Psychiatrist 5)

*“So having the time to do that, the time and space to give your attention to a person with schizophrenia because sometimes cognitively, or because of some issues they might have with executive functioning, you do need to just have that extra time with them, looking at different ways to communicate.”*

(Pharmacist 8)

The nature of patient’s illness and patient acceptability of community pharmacists’ monitoring of cardiometabolic adverse events was also a concern for participants.

*“Going out to a pharmacy may be a bit restrictive, especially with schizophrenia, with negative symptoms, lack of motivation, those kinds of things. Maybe a bit of a hindrance for people to go out to the pharmacy because you’re leaving your comfort zone.”*

(Psychiatrist 5)

*“Some of them have got paranoia and things like that, so it that could be a complicating factor really. If the patients didn’t feel comfortable with that occurring, you wouldn’t be able to do it [monitoring]”.*

(Pharmacist 1)

Acceptance from other healthcare professionals, such as general practitioners, was raised by participants as a possible concern.

*“…I see that, as you know, sadly a bit of a turf war, and I think that could be a barrier. But I think it does depend on the GP and the relationship that a pharmacist does have with them.”*

(Pharmacist 8)

Some participants thought that the inclusion of community pharmacists in a multidisciplinary mental healthcare team was unnecessary.

*“I think from a cardiometabolic point of view, for a community mental health team that I work in, I think there is a risk of that information getting, uhm, lost in the communication pathway. I think there’s a risk of too many cooks in the kitchen.”*

(Pharmacist 2)

Most participants found that having community pharmacists involved would help to reduce the workload of the team.

*“I think the workload in mental healthcare, in general, is just skyrocketing everywhere for everyone…I dare say it would prevent adverse events from happening down the track for a lot of people.”*

(Nurse 9)

*“At the end of the day, it’s a mental health service, so you concentrate more on psychotic symptoms and (anti) psychotic medication… that they are not suicidal, they’re not homicidal, so to be able to actually focus on the mental health problems of the patients… it would be great…So if the pharmacist is focusing more on the physical and metabolic health of the patient, it would allow the nurses and the clinic staff to focus on that mental health.”*

(Nurse 6)

## 4. Discussion

To our knowledge, this was the first Australian study to explore the opinions of mental health professionals on the role of community pharmacists in monitoring and managing the incidence of cardiometabolic adverse drug events in people with schizophrenia. According to this study, mental health professionals believed there were established benefits to including community pharmacists in the collaborative management of people with schizophrenia, and that community pharmacists have the capability to monitor cardiometabolic parameters and reduce the occurrence of cardiometabolic adverse drug events. However, some barriers to their integration into a multidisciplinary mental health team were identified.

Mental health professionals viewed community pharmacists as capable of delivering professional services, including education and counselling, conducting medication reviews, and providing recommendations to healthcare professionals for people with schizophrenia. Based on the experiences of mental health professionals, they referred to several community pharmacist-led intervention programs that have shown clinical benefits to patients in areas such as asthma management, diabetes control, hypertension and cardiovascular disease, weight management, and smoking cessation [15,22,23]. The participants in this study were confident in the qualifications and resources of community pharmacists for monitoring cardiometabolic parameters such as blood pressure, weight, and waist circumference.

As reported by mental health professionals, if community pharmacists were to have a greater role in cardiometabolic parameter monitoring for people with schizophrenia, this could include point-of-care testing for lipids and blood glucose, with some participants suggesting this could perhaps be extended to include the provision of pathology referrals or services. A study by Donovan et al. reviewed the scope of practice for community pharmacists’ regarding accessing and/or ordering pathology tests in countries, including Canada, the USA, the UK, Australia, and New Zealand [24]. Access to patient records and the ability to refer for laboratory tests differed between countries and even states, often guided by specific guidelines and practice agreements. The systematic review indicated positive patient outcomes, including reduced cardiovascular risk, lower blood pressure and improved lipid control.

To improve medication compliance in people with schizophrenia, participants in this study suggested that community pharmacists could potentially administer antipsychotic depot medications, a practice already trialled at some sites internationally [25]. Recent studies have demonstrated an increased adherence with community pharmacists administering long-acting injectable antipsychotics [26,27]. However, this approach has not been trialled nor implemented in Australia.

Participants articulated the importance of communication between community pharmacists, mental health teams and general practitioners (GPs), with previous studies underscoring the necessity for improved interdisciplinary communication among mental healthcare providers [28]. Particularly, mental health professionals expressed the significance of enhancing collaboration between community pharmacists and GPs, to ensure that GPs accept the role of community pharmacists within a multidisciplinary team. Despite both GPs and community pharmacists acknowledging the importance of a therapeutic alliance for patients with chronic conditions, studies have shown collaboration between the disciplines is limited in many areas of medicine, including in the management of people with mental illness [29,30,31]. In addition, studies have suggested that GP’s perceptions of the value of community pharmacist collaboration could be influenced by factors such as existing relationships, encroachment on professional boundaries and the need for clearly defined roles, perceived conflict of interest on the pharmacist’s part, the continuity of pharmacy staff, and the territoriality and hierarchy within the GP profession [29,30,31,32]. Notably, our findings reinforce the potential benefits to people with schizophrenia, of better collaboration with mental health professionals and GPs, perhaps resulting in earlier intervention for adverse drug events such as hyperlipidaemia, hypertension, and hyperglycaemia.

The proposal of establishing a formal communication pathway or leveraging electronic resources like My Health Record (MHR) in Australia, was put forward to address this. Community pharmacists’ utilization of patients’ MHR has the potential to mitigate the “fragmentation of care” [33]. A survey of Australian pharmacists in 2018 identified that reducing medication errors and enhancing collaboration with other healthcare professionals were benefits associated with the use of MHR, aligning with overseas experiences where the utilization of patients’ electronic medical records resulted in reduced hospitalizations [34,35].

In line with the views of mental health experts, while community pharmacists receive sufficient training to monitor cardiometabolic parameters, mental health first aid (MHFA) training may not be comprehensive enough. Advanced mental health training beyond MHFA, including role plays and interaction with mental health consumer educators, has been shown to improve community pharmacists’ knowledge and confidence in screening for, and intervening in, cardiometabolic adverse events in people with severe mental illness [36,37].

According to the narratives of those in the mental health field, a role in a multidisciplinary team and the provision of cardiometabolic monitoring services for people with schizophrenia would have to be acceptable to community pharmacists themselves. An Australian interventional study into mental health medication management in community pharmacies found that 45% of participating pharmacies were unsuccessful in delivering interventions, with the lack of success attributed to inadequate support from pharmacy owners or managers, insufficient buy-in from pharmacists, inadequate staffing levels, and time constraints [38].

Based on participants’ insights, acceptability by people with schizophrenia could pose a barrier to the inclusion of community pharmacists in a multidisciplinary team. However, studies have shown that many patients are comfortable discussing mental health issues with community pharmacists who have undergone MHFA training, identifying the importance of the use of language that implies to the patient, a close collaboration with other mental healthcare professionals [39,40].

Furthermore, based on the experiences of mental health professionals, cardiometabolic parameter monitoring is often performed by allied health staff, such as occupational therapists or psychologists, who may lack training in using equipment for monitoring or interpreting results. A UK study found that a third of mental health professionals were uncertain about who should be responsible for monitoring the physical health of a person with schizophrenia (a psychiatrist or a GP), and more than 50% were uncertain as to how to interpret abnormal cardiometabolic parameter results [41]. Mental health professionals in this study suggested that involving community pharmacists in monitoring cardiometabolic parameters could help alleviate the workload of mental health teams.

The strength of this study lies in its ability to capture diverse perspectives from mental health professionals with varying years of experience, allowing them to share their thoughts and beliefs regarding the role of community pharmacists in managing people with schizophrenia. A limitation, however, is that it did not include the opinions of GPs. Given the pivotal role GPs play in the physical health of people with schizophrenia, future research could benefit from seeking their perspectives on the involvement of community pharmacists in monitoring and managing adverse cardiometabolic events in these patients, as well as their inclusion in multidisciplinary mental health teams. Additionally, it’s worth noting that the participants in this study were from New South Wales and South Australia, hence, the opinions of mental health professionals may only reflect the practices in these states.

## 5. Conclusions

In conclusion, mental health professionals perceive community pharmacists as having the training and resources necessary for metabolic parameter monitoring, however, they recommend further specialized training for managing people with severe mental illnesses. Although mental health professionals acknowledged the benefits of integrating community pharmacists into multidisciplinary teams, they also recognized certain perceived barriers to their inclusion. These barriers encompass concerns about the need for remuneration, availability of staff resources, time constraints, acceptance by other healthcare professionals and patients, and the necessity for improved communication pathways. Addressing these barriers and providing targeted training could facilitate the valuable inclusion of community pharmacists in the comprehensive care of people with schizophrenia.

## Figures and Tables

**Table 1:** Participant demographics (*n* = 11).

Participant	Gender	Age Distribution	Profession	Years of Experience Practicing Mental Health	Australian State or Territory *	Location	Interview Duration (Minutes)
1	Female	56–65	Pharmacist	36–45	NSW	Regional	33
2	Female	36–45	Pharmacist	6–15	SA	Metropolitan	34
3	Female	56–65	Pharmacist	16–25	NSW	Metropolitan	30
4	Female	26–35	Psychiatrist	0–5	NSW	Metropolitan	18
5	Male	46–55	Psychiatrist	16–25	NSW	Regional	23
6	Female	66+	Nurse	36–45	NSW	Metropolitan	27
7	Female	56–65	Nurse	36–45	NSW	Metropolitan	27
8	Female	46–55	Pharmacist	16–25	NSW	Regional	37
9	Male	26–35	Nurse	6–15	NSW	Metropolitan	26
10	Female	46–55	Psychiatrist	16–25	NSW	Metropolitan	35
11	Female	36–45	Pharmacist	16–25	NSW	Regional	33

* NSW = New South Wales; SA = South Australia.

**Table 2:** Themes and sub-themes.

Themes and Sub-Themes
Theme 1: The benefits of community pharmacists’ involvement in the management of cardiometabolic adverse drug events in people with schizophrenia.
1.1. Provision of professional services from community pharmacists currently
1.2. Positive rapport with patients
1.3. Accessibility of community pharmacies
1.4. Potential to extend professional services with current pharmacist competencies
Theme 2: Improving communication pathways with community pharmacists
2.1. No established communication pathway for feedback to mental health professionals
2.2. Under-utilization of My Health Record (MHR)
2.3. No access to patients’ legal records.
Theme 3: Defining the role and responsibility for monitoring cardiometabolic parameters and managing adverse cardiometabolic drug events.
3.1. No consensus on responsibility for monitoring cardiometabolic parameters
3.2. No consensus on responsibility for managing cardiometabolic adverse events
3.3. Community pharmacists may assist early intervention for adverse cardiometabolic events
3.4. Community pharmacists’ role necessitates further mental health training
Theme 4: Fostering collaboration between community pharmacists and mental health care professionals.
4.1. Collaboration with community pharmacists beneficial for mental health professionals
4.2. Importance of collaboration with general practitioners
4.3. Well-defined pathway to feedback metabolic results required
Theme 5: Recognizing the acceptance of community pharmacists’ involvement in a multidisciplinary team.
5.1. Acceptance of community pharmacists within the team
5.2. Acceptance of community pharmacists by people with schizophrenia
5.3. Acceptance of community pharmacists by other healthcare professionals

## Data Availability

The de-identified transcripts analysed for the current publication are available from the corresponding author upon reasonable request.

## Supplementary Materials

The following supporting information can be downloaded at: https://www.mdpi.com/article/10.3390/medicina59122052/s1, File S1: Interview guide.
