# Supplementary material for: Community Pharmacists’ Role in Reducing the Incidence of Cardiometabolic Adverse Drug Events in Schizophrenia: Insights from Mental Health Professionals
Source: Medicina (Kaunas). 2023 Nov 21;59(12):2052. doi: 10.3390/medicina59122052 (PMC10744378; doi:10.3390/medicina59122052)
Supplement: Supplementary file 1 [file medicina-59-02052-s001.zip › medicina-2695006-supplementary.pdf]

Supplementary File S1. Interview Guide

Title: Community Pharmacists' Role in Reducing the Incidence of Cardiometabolic Adverse Drug Events in Schizophrenia: Insights from Mental Health Professionals

Semi-structured interviews will use open and expansive questions; however, we will need to be flexible and ask follow-up questions if warranted.

Introduction:

- Introduce myself and confirm the participants' details
- The interview will take approximately 30 minutes and will be recorded for transcription
- The transcripts will be de-identified, and they will not be individually identifiable in any research outputs
- Participants can pause, stop, or withdraw from the interview and study at any point
- Ask if the participant has any questions or if they require further explanation before we begin

Participant details:

- Confirm demographic data obtained on the consent form
- Confirm their role and how long they have been in the current role
- Ascertain their geographical area of practice (metropolitan, regional, or remote).

We would like to discuss your perspective on community pharmacists managing cardiometabolic adverse effects.

Healthcare professional's perspective on collaboration with community pharmacists regarding the management of schizophrenic patients:

- Could you tell me of a time when a community pharmacist has positively influenced the care of a patient with schizophrenia? What did the pharmacist do? How do you know there was a positive outcome? What interactions with the health care team occurred?
- Could you tell me of a time when an intervention by a community pharmacist has had a negative effect on the care of a patient with schizophrenia? What did the pharmacist do? How do you know it was a negative outcome? What could the pharmacist have done to improve the interaction? What interactions with the healthcare team occurred?
- If neither good/nor bad/nor any recall: Why do you think that you cannot think of any positive or negative experiences with community pharmacists? How could community pharmacists collaborate more with the health care team and with patients?

The capacity of pharmacists to do monitoring and feedback in the community pharmacy (resources and barriers):

- In your opinion, how beneficial are community pharmacists in the management of patients with schizophrenia? Why do you say that? / What influenced your opinion?

- How would you like community pharmacists to be involved in the management of patients with schizophrenia? (If not answered above)
- Do you feel that community pharmacists are capable of monitoring cardiometabolic parameters in community pharmacies? Why do you feel that?
- What resources or skills do you think pharmacists require to successfully manage cardiometabolic adverse events (e.g. information/training)?
- What resources or skills do you believe pharmacists currently have?
- Do you see any barriers to community pharmacist involvement in managing cardiometabolic adverse events in schizophrenic patients?

What is the attitude of mental healthcare professionals on the inclusion of community pharmacists in a multidisciplinary team, to deliver a cardiometabolic parameter monitoring service for patients with schizophrenia?

- Do you feel there is a benefit in including community pharmacists in community mental health teams regarding the management of patients with mental illnesses such as schizophrenia? Why?
- What is your attitude toward community pharmacist interventions in addressing adverse cardiometabolic drug events?
- What is your perception of how your patients with schizophrenia will respond to community pharmacist interactions?
- What do you see as the barriers to community pharmacist involvement in the successful management of patients with schizophrenia? How might they be overcome?

Conclusion:

- That concludes the questions I wanted to ask. Do you have any final comments relating to what we have discussed? Is there an area related to community pharmacist involvement in the management of patients with schizophrenia that you would like to discuss?
- Thank you.
- Confirm if the participant would like to review the transcript of their interview (as per the consent form).
- Confirm participant’s details for sending voucher (if remunerating participants).
- Confirm if they would like to be kept up to date with the research outcomes (this will also be on the consent form).
